# Clinical outcomes and quality of life associated with the use of a biosynthetic mesh for complex ventral hernia repair: analysis of the “Italian Hernia Club” registry

**DOI:** 10.1038/s41598-020-67821-w

**Published:** 2020-07-01

**Authors:** Carla Rognoni, Diego Cuccurullo, Ludovica Borsoi, Luigi Bonavina, Emanuele Asti, Feliciano Crovella, Uberto Andrea Bassi, Gabriele Carbone, Francesca Guerini, Paolo De Paolis, Silvia Pessione, Vincenzo Maria Greco, Elisabetta Baccarini, Giorgio Soliani, Carlo Sagnelli, Clotilde Crovella, Vincenzo Trapani, Carlo De Nisco, Emilio Eugeni, Federico Zanzi, Enrico De Nicola, Antonio Marioni, Alessandro Rosignoli, Roberto Silvestro, Rosanna Tarricone, Micaela Piccoli

**Affiliations:** 1 Centre for Research on Health and Social Care Management (CERGAS), SDA Bocconi School of Management, Bocconi University, Milano, Italy; 2 Ospedale Monaldi, Azienda Ospedaliera dei Colli, Napoli, Italy; 3 IRCCS Policlinico San Donato, San Donato Milanese, Milano, Italy; 4 CTO, Azienda Ospedaliera dei Colli, Napoli, Italy; 5 Poliambulanza, Brescia, Italy; 6 Città della Salute, Ospedale Molinette, Torino, Italy; 7 Week Surgery, Policlinico S. Orsola-Malpighi, Sede di Budrio, Bologna, Italy; 8 Azienda Ospedaliero Universitaria, Ferrara, Italy; 9 Azienda Ospedaliero-Universitaria, OCB (Ospedale Civile Baggiovara), Modena, Italy; 10 Ospedale San Francesco, Nuoro, Italy; 11 Presidio Ospedaliero San Salvatore, Pesaro, Italy; 12 AUSL della Romagna, Ospedale S. Maria delle Croci, Ravenna, Italy; 13 Ospedale San Paolo, Milano, Italy; 14 AO Universitaria Pisana, Pisa, Italy; 15 Ospedale Santa Maria della Misericordia, Udine, Italy; 16 Department of Social and Political Sciences, Bocconi University, Milano, Italy

**Keywords:** Materials for devices, Quality of life, Outcomes research

## Abstract

With the development of newer meshes and approaches to hernia repair, it is currently difficult to evaluate their performances while considering the patients’ perspective. The aim of the study was to assess the clinical outcomes and quality of life consequences of abdominal hernia repairs performed in Italy using Phasix and Phasix ST meshes through the analysis of real-world data to support the choice of new generation biosynthetic meshes. An observational, prospective, multicentre study was conducted in 10 Italian clinical centres from May 2015 to February 2018 and in 15 Italian clinical centres from March 2018 to May 2019. The evaluation focused on patients with VHWG grade II–III who underwent primary ventral hernia repair or incisional hernia intervention with a follow-up of at least 18 months. Primary endpoints included complications’ rates, and secondary outcomes focused on patient quality of life as measured by the EuroQol questionnaire. Seventy-five patients were analysed. The main complications were: 1.3% infected mesh removal, 4.0% superficial infection requiring procedural intervention, 0% deep/organ infection, 8.0% recurrence, 5.3% reintervention, and 6.7% drained seroma. The mean quality of life utility values ranged from 0.768 (baseline) to 0.967 (36 months). To date, Phasix meshes have proven to be suitable prostheses in preventing recurrence, with promising outcomes in terms of early and late complications and in improving patient quality of life.

## Introduction

Abdominal hernia represents the protrusion of a viscera from the cavity that normally contains it through an orifice or a pre-existing or new-onset anatomical channel. Abdominal hernia represents an extremely common disease with a variable incidence that ranges from 4 to 8% per year^1–3^, that mainly affects the male gender, particularly in cases of inguinal hernia, which are 8–10 times more common in men than in women, and peaks between 40 and 60 years^4–6^. Abdominal hernias represent a benign pathology with a potential progressive trend in terms of volume increase and the appearance of symptoms of entrapment or strangulation of the herniated bowels such as nausea, vomiting or greater symptoms from peritonitis (perforation of the herniated bowel) which can require urgent treatment.

Abdominal hernia can be primary or acquired: the first group includes primary ventral hernias (midline as epigastric and umbilical, and lateral as Spigelian and lumbar), while incisional hernias are considered as acquired forms on previous surgical incisions^7^.

Any hernia must be surgically treated to be corrected, and surgery is the only way to reconstruct the abdominal/inguinal wall. Reparative techniques can be performed with the use of prosthetic (alloplastic) materials that can be made of synthetic (polypropylene, polyester, PTFE- and composite) or biological (porcine dermis, bovine pericardium) material. The use of biocompatible prosthetic materials, which began in the 1990s, was supported by certain undoubted advantages linked both to their tensile capacity, which mechanically supports the dynamism of the abdominal wall in the initial phases, and their ability to integrate into the tissues, stimulating a biological reaction from the host. Recently, biosynthetic meshes have been proposed as a new class of materials. Derived from biological sources (human, bovine, or porcine) or using absorbable synthetic material, these meshes are theoretically incorporated into native tissue and show the ability to resist to infections^8^.

Among biosynthetic meshes, Phasix (Bard/Davol) is a macroporous, fully absorbable synthetic mesh that consists of co-knitted absorbable poly-4-hydroxybutyrate, while Phasix ST (Bard/Davol) is a composite mesh with additional polyglycolic acid fibres coated with a chemically modified sodium hyaluronate, carboxymethylcellulose, and polyethylene glycol-based hydrogel on the visceral surface, which allows intraperitoneal positioning^8–11^. In preclinical testing, both of these products demonstrated high tensile strength and flexibility^12^ with increased repair strength noted during tissue remodelling^13^.

While the variety of materials and surgical techniques give the surgeon the opportunity to perform a tailored surgery, this variety also makes it is difficult to evaluate the performance of the different prostheses in the diverse surgical contexts while also considering the patients’ perspective, and there is paucity of studies reporting data on the perceived quality of life (QoL) before and after hernia repair. In this context, the analysis of real-world data collected through national registries combined with data reported in the literature may be advisable to assess the performance of different types of prostheses.

At the international level, different countries (Sweden, Denmark, Germany, Austria and Switzerland, France, Spain and USA) are developing databases for the collection of outcome variables following hernia surgery^14^. In general, the aim of these registries is to report on the operative techniques used and to analyse outcome measures to achieve quality improvement. The recorded data mainly include the patients’ personal data, risk factors and comorbidities, surgical routes (ventral, inguinal, parastomal, incisional, etc.), pre-operative data collection, operating time, type of mesh, concomitant therapy, intra-operative/post-operative complications (mesh infection, mesh removal, etc.), post-surgical pain, QoL and mortality.

In Italy, the “Italian Hernia Club” multicentre registry was founded in 2015 to collect data on Phasix meshes; data collection began in ten clinical centres. The registry is currently collecting specific data on hernia surgeries and on patients’ follow-up, including the assessment of their QoL over time. These data have already been used to compare the current economic impact of the management of patients with complex incisional abdominal hernia through biosynthetic mesh implants versus synthetic or biologic meshes, from the hospital perspective in Italy^15^.

The aim of the present study was to analyse the clinical outcomes and QoL consequences of hernia repairs using Phasix and Phasix ST mesh products performed in Italy to add evidence to support the choice of new generation biosynthetic prostheses. The evaluation considered different aspects of the management of patients undergoing hernia repair, including the management of different complications.

## Methods

### Study design

The “Italian Hernia Club” registry is an observational, prospective, multicentre study that began in 10 Italian clinical centres in May 2015 and has since been extended to a total of 15 Italian centres as of March 2018. The registry collects data from adult patients undergoing abdominal hernia repair with Phasix (extraperitoneal positioning) or Phasix ST (intraperitoneal positioning) prostheses only, at the centres participating in the study. At the cut-off date of 13 June 2019, the registry contained data on 275 patients.

The present analysis focused on data collected until 13 June 2019 and considered the selection of adult patients with diagnosis of abdominal hernia who were risk grade II or III according to the Ventral Hernia Working Group classification^16^ and had a follow-up of at least 18 months. Because Phasix meshes are absorbable within 12–18 months^17^, a constraint on the follow-up duration was applied to perform an evaluation after prosthesis absorption.

Electronic medical records accessible via the web were completed by the clinicians at the clinical centres involved to record all the data related to the preintervention visit, the intervention itself and the follow-up visits (predefined timing at 8 days, 30 days, 6–12–18–24–36–48–60 months). The follow-up was performed during clinical evaluations (outpatient visits with physical examination) in the first month after the intervention and with telephone follow-up in the subsequent months. In cases of suspected relapse or complications, telephone follow-up is associated with an outpatient visit; if necessary, imaging examinations (ultrasound, CT scan) and blood tests are performed.

The study protocol has been approved by the Ethics Committee of Bocconi University and of each clinical centre involved (Ospedale Monaldi, Azienda Ospedaliera dei Colli, Napoli; IRCCS Policlinico San Donato, San Donato Milanese, Milano; CTO, Azienda Ospedaliera dei Colli, Napoli; Poliambulanza, Brescia; Città della Salute, Ospedale Molinette, Torino; Week Surgery, Policlinico S. Orsola-Malpighi, Sede di Budrio, Bologna; Azienda Ospedaliero Universitaria, Ferrara; Azienda Ospedaliero-Universitaria, OCB (Ospedale Civile Baggiovara), Modena; Ospedale San Francesco, Nuoro; Presidio Ospedaliero San Salvatore, Pesaro; AUSL della Romagna, Ospedale S. Maria delle Croci, Ravenna; Ospedale San Paolo, Milano; AO Universitaria Pisana, Pisa; Ospedale Santa Maria della Misericordia, Udine); the study protocol conformed with the Helsinki Declaration of 1964, as revised in 2013, concerning human and animal rights. An informed consent has been obtained from all participants and/or their legal guardians.

### Outcomes

The collected data included personal data (gender, date of birth), anamnesis (height, weight, BMI, risk class), diagnosis (hernia type, information on pre-operative imaging), intervention (date, type, scheme, duration of hospitalisation), presence of drains, prosthesis used (Phasix or Phasix ST) and prosthesis characteristics. The follow-up investigated, as the primary outcome, the rates of complications including recurrence, infected mesh removal, superficial infection, deep infection, organ infection, and seroma. During the follow-up, the assessment of a secondary outcome related to QoL was performed according to the EuroQol 5-dimension 5-level questionnaire (EQ-5D-5L)^18^.

The EQ-5D-5L is a standardized tool that allows measuring the QoL of the respondents. This questionnaire has an estimated completion time is approximately 5 min and consists of two distinct sections. The first section is a descriptive system containing 5 questions to investigate the patient’s perceived health status on five dimensions: mobility, self-care, usual activities, pain/discomfort and anxiety/depression. Each dimension has 5 levels, from no problems to extreme problems. The second section includes a visual analogue scale (VAS), which is graphically represented as a thermometer graduated from 0 (worst possible health state) to 100 (best possible health state) and on which the interviewee indicates the perceived level of his/her health status. An algorithm allows the calculation of a final score (utility coefficient) based on the assignment of weights to the health states obtained through the descriptive system. The utility coefficient can take on values that range from 1 (perfect health) to negative values for health states worse than death (0). A higher score corresponds to a better state of health. In the present study, the utility weights for the English population were used^19^, as the EQ-5D-5L value set is currently not available for the Italian population.

The data recorded in the Italian Hernia Club registry were mainly abstracted from the case report forms, in both electronic or paper archive form; this concerns the hospital stay, interventions, exams and follow-up. The responses to the EQ-5D-5L questionnaire were recorded directly into the registry.

### Statistical analysis

Numerical variables were summarized using means, standard deviations, and 95% confidence intervals (CIs). Categorical variables were summarized using counts and percentages. The evaluation of differences between groups was performed by applying the T test or Wilcoxon test (continuous variables) or chi-square/Fisher tests (proportions). Linear mixed-effects models, which account for correlated (within-patient) data, were used to evaluate changes in patients’ QoL over time. Overall recurrence rates were projected using Kaplan–Meier estimation and 95% confidence intervals. For all testing, a p value < 0.05 was considered statistically significant. Statistical analyses were performed using STATA software (version 15.0, https://www.stata.com).

## Results

### Patient characteristics and clinical outcomes

A total of 75 patients reporting VHWG grade II or III who underwent primary ventral hernia or incisional hernia repair with a follow-up of at least 18 months were included in the Italian Hernia Club registry on 13th June 2019. Table 1 reports the patients’ characteristics.

**Table 1:** Summary of patient demographics and comorbid conditions.

Age, years (range)	59 (30–87)
Gender (male), n (%)	35 (47%)
Body mass index, kg/m^2^ (range)	30 (18–61)
**Comorbid conditions, n (%)**	
Previous associated infections	22 (29%)
Previous interventions	53 (71%)
Obesity (BMI > 30)	26 (35%)
Active smoking	26 (35%)
Diabetes mellitus	17 (23%)
Chronic obstructive pulmonary disease	17 (23%)
Immunosuppression	7 (9%)

The included patients had a mean follow-up of 792 days (± 191 days), a mean age of 59 years, a BMI of 30 and were 47% men. Forty-three patients (57.33%) arrived at the intervention with no hernia recurrence, while the remaining were at one (32.00%), two (5.33%) or three or more (5.33%) recurrences (see Table 2 for details on operative characteristics). The mean duration of the intervention was 185 min, and in most cases the repair was performed by Transversus Abdominis Release (TAR) (33.3%), Rives–Stoppa (30.7%), open IntraPeritoneal Onlay Mesh (IPOM) (17.3%) or external oblique release (12.0%); the intervention was performed electively in 80% of patients. As regards the positioning, in most cases the mesh was set in retrorectus/preperitoneal seat (73%), followed by intraperitoneal (19%) and onlay (4%) locations. The mean hospitalisation length was 11 days (range 1–37 days, 10 days for election, 13 days for emergency). Phasix meshes (73%) were implanted more frequently than Phasix ST prostheses (27%). Absorbable sutures were used in 66% of cases, while in the remaining cases, a non-absorbable suture was applied. Hernia widths of < 5 cm, 5–10 cm, 10–15 cm, and > 15 cm were reported in 13%, 51%, 23% and 13% of cases, respectively, while the same figures for hernia length were 5%, 43%, 23% and 29%, respectively. The mean dimension of the prostheses was 658 cm^2^ (range 70–3,020).

**Table 2:** Operative characteristics: hernia defect and mesh dimensions, surgical approach, wound and hernia defect characteristics.

**Wound characteristics**	
VHWG grade II, n (%)	40 (53%)
VHWG grade III, n (%)	35 (47%)
**Hernia defect characteristics**	
Defect size, mean (range)	431 cm^2^ (10–2,190)
Defect length, mean (range)	21 cm (5–45)
Defect width, mean (range)	20 cm (2–146)
Mesh dimension, mean (range)	658 cm^2^ (70–3,020)
Surgical procedure time, mean (range)	185 min (45–540)
**Surgical technique, n (%)**	
Rives–Stoppa	23 (30.7%)
Transversus abdominis release (TAR)	25 (33.3%)
External oblique release	9 (12.0%)
Open intraperitoneal onlay mesh (IPOM)	13 (17.3%)
Endoscopic repair	1 (1.3%)
Laparoscopic repair	1 (1.3%)
Not reported	3 (4.0%)
**Mesh positioning, n (%)**	
Intraperitoneal	14 (19%)
Onlay	3 (4%)
Retrorectus/preperitoneal	55 (73%)
Not reported	3 (4%)
Recurrent hernia repaired, n (%)	32 (42.7%)
**Type of hernia, n (%)**	
Incisional hernia	53 (70.7%)
Incisional hernia with other concomitant procedures	15 (20.0%)
Primary ventral hernia	1 (1.3%)
Primary ventral hernia with other concomitant procedures	1 (1.3%)
Parastomal hernia	1 (1.3%)
Parastomal hernia with other concomitant procedures	4 (5.3%)
**Hernia location**	
Single location, n (%)	55 (73%)
Midline hernias:	
Subxiphoidal	7 (12.7%)
Epigastric	13 (23.6%)
Umbilical	17 (30.9%)
Infraumbilical	6 (10.9%)
Suprapubic	2 (3.6%)
Lateral hernias:	
Subcostal	1 (1.8%)
Flank	1 (1.8%)
Iliac	8 (14.5%)
Lumbar	–
Multiple locations, n (%)	20 (27%)
**Concomitant procedures, n (%)**	20 (27%)
Colostomy	2 (10%)
Colostomy reversal	1 (5%)
Bowel resection plus colostomy	1 (5%)
Bowel resection	10 (50%)
Rectal resection	1 (5%)
Liver resection/segmentectomy	2 (10%)
Spleno-pancreatic resection	1 (5%)
Fistula removal	1 (5%)
Uretero-enterostomy	1 (5%)

Thirteen patients (17%) reported a superficial infection, but only three of them (4%) had surgical site occurrences requiring procedural interventions^20^. Four of the 13 superficial infections have been reported for Phasix ST (intraperitoneal) and in two cases a procedural intervention has been performed. One Phasix mesh (1.3%) placed in the epigastric seat was removed (partially) because of infection 7 days after the intervention in a patient with comorbid conditions (smoker, BPCO). In this study population, no patients reported deep or organ infections.

**Figure 1:**
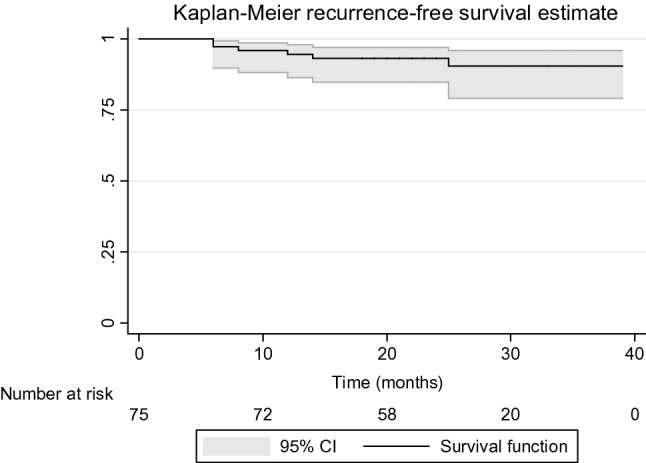
Kaplan–Meier recurrence-free survival estimate.

Six patients (8%) had a recurrence at a mean time of 357 days (range 176–755) after the intervention. The mean follow-up of these patients was 709 days (nearly 24 months). Four of them (67%) were VHWG grade 2, and two of them (33%) were VHWG grade 3. No correlation between wound complications and recurrence was observed (Fisher test p = 0.679). Figure 1 shows the recurrence-free Kaplan–Meier curve. Recurrences have been reported after IPOM surgery in 3 cases (50%), after Rives–Stoppa intervention in one case (17%), and after TAR and laparoscopic surgery in the other two patients (33%). All these patients except one underwent the intervention electively. Among retrorectus and preperitoneal interventions (n = 55) only 2 cases of recurrence were found. In patients who had a recurrence, the mean prosthesis area was larger (1,003 cm^2^, range 300–3,020) than in patients without recurrence (628 cm^2^, range 70–2,500).

A seroma was reported in 13 patients (17%), and in 5 of them (6.7%), percutaneous drainage was required.

Four patients underwent a subsequent intervention (5.3%) for the following causes: compartment syndrome, mesh substitution after infected (partial) mesh removal, fistula removal and development of a new incisional hernia (epigastric).

A summary of the complication rates is reported in Table 3.

**Table 3:** Primary outcomes.

**Complications, n (%)**	
Recurrence	6 (8.0%)
Infected mesh removal	1 (1.3%)
Superficial infection requiring procedural intervention	3 (4.0%)
Deep/organ infection	–
Seroma requiring percutaneous drainage	5 (6.7%)
Re-intervention	4 (5.3%)

### Patient-reported quality of life

The administration of the EQ-5D-5L questionnaires started on 2 March 2017, and for this reason, data on patients’ quality of life was not available for all the patients under study; indeed, it was only possible to estimate the utility values related to the preintervention visit (baseline) in 56 out of 75 patients.

**Figure 2:**
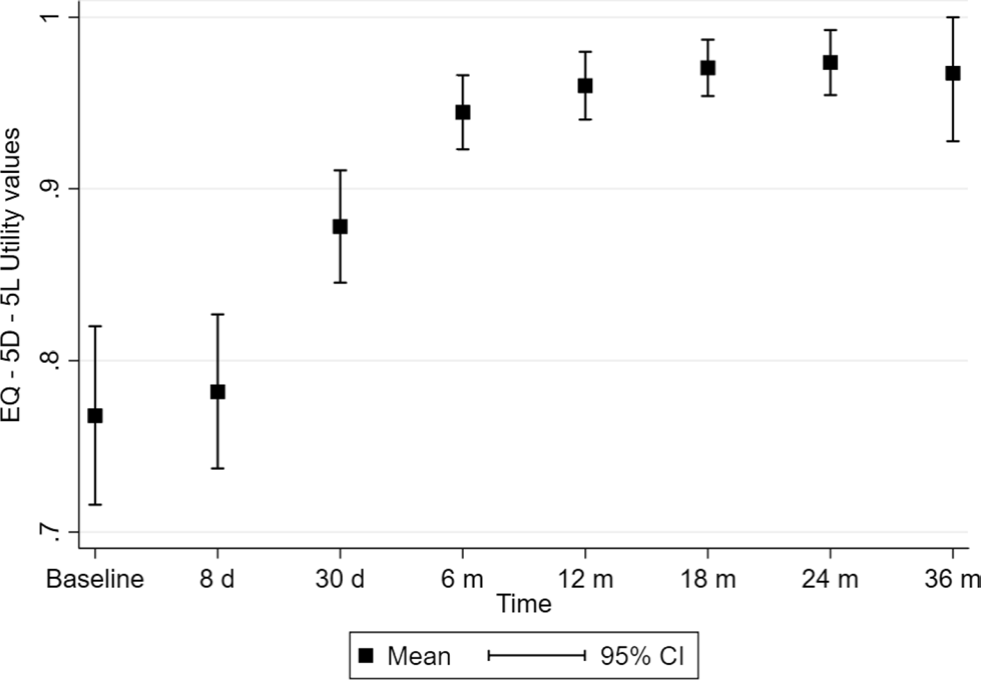
Change of EQ-5D-5L utility values over time (*d* days, *m* months).

Patients reported a substantial improvement in quality of life over the follow-up period (Fig. 2). With the exception of the measure at 8 days postoperatively, the increase from baseline on the EQ-5D-5L utility values was statistically significant (p < 0.001) at all follow-up time points (Table 4). Moreover, the percentage of patients reporting perfect health (i.e. % ceiling) substantially increased over time (from 19.6% at baseline to 80% at 36 months). Stratification of the patients according to VHWG risk classification revealed that the QoL of grade II patients was not significantly different from that of grade III patients during the follow-up period, with the only exception of month 18, when grade II patients showed a significantly lower utility value (0.957 vs. 0.985, T test p < 0.05) compared to grade III patients. Overall, QoL was lower in patients who experienced infections or recurrences (baseline value 0.729, 36-month value 0.950). Table 5 shows that a statistically significant improvement in all EQ-5D-5L dimensions was observed over time. Specifically, patients reported a sustained decrease in pain, lower levels of anxiety and depression and an increased ability to carry out usual activities with respect to baseline.

**Table 4:** Descriptive statistics of EQ-5D-5L utility values from Italian Hernia Club registry.

Time	N	Mean	Std error	95% CI	% floor	% ceiling	p*
Baseline	56	0.768	0.026	0.716–0.820	0.0	19.6	–
8 days	57	0.782	0.022	0.737–0.827	0.0	19.3	0.431
30 days	59	0.878	0.016	0.845–0.911	0.0	32.2	< 0.001
6 months	60	0.945	0.011	0.923–0.966	0.0	56.7	< 0.001
12 months	60	0.960	0.010	0.940–0.980	0.0	66.7	< 0.001
18 months	63	0.971	0.008	0.954–0.987	0.0	76.2	< 0.001
24 months	52	0.974	0.009	0.955–0.993	0.0	78.8	< 0.001
36 months	20	0.967	0.019	0.928–1.000	0.0	80.0	< 0.001

% floor refers to the percentage of patients reporting the lowest utility score (i.e. − 0.285). % ceiling refers to the percentage of patients reporting the highest utility score (i.e. 1);

*All p values refer to the change in mean utility with respect to baseline.

**Table 5:** Mean change in EQ-5D-5L dimension scores from baseline (data from Italian Hernia Club registry).

EQ-5D-5L dimensions	Baseline (mean)	Mean change from baseline						
		8 days	30 days	6 months	12 months	18 months	24 months	36 months
Movement	1.6	0.1	− 0.3***	− 0.6***	− 0.5***	− 0.5***	− 0.6***	− 0.6***
Self-care	1.5	0.2*	− 0.3***	− 0.5***	− 0.5***	− 0.5***	− 0.5***	− 0.4***
Usual activities	1.9	0.0	− 0.5***	− 0.8***	− 0.8***	− 0.8***	− 0.8***	− 0.8***
Pain/discomfort	2.3	− 0.3**	− 0.8***	− 1.1***	− 1.1***	− 1.1***	− 1.2***	− 1.2***
Anxiety/depression	2.1	− 0.2*	− 0.4***	− 0.6***	− 0.8***	− 0.9***	− 0.8***	− 0.8***

The EQ-5D-5L dimensions can take on values from 1 (no problems) to 5 (extreme problems).

*p < 0.05, **p < 0.01, ***p < 0.001.

## Discussion

In recent years, the context of hernia repair has witnessed many changes, starting from the minimally invasive laparoscopic surgical approach to the introduction of different types of prostheses. Among them, there was a shift from autogenous tissue repair to the use of synthetic, biosynthetic and biological meshes. The main goals of surgery are optimal repair, reduction of pain, quick recovery and low recurrence. The surgeons may choose from many meshes and surgical approaches/fixation techniques, and each of them has its own advantages and disadvantages. This freedom of choice allows surgeons to select the most appropriate treatment for each individual patient to achieve the best outcomes. Unfortunately, it is not easy to identify the best option for each specific patient, especially while taking into account that the literature also reports debates on this issue. For example, a few review articles^21–23^ highlighted the issue of the poor reporting standards of studies on the use of biologic meshes for different abdominal wall repairs. The controversial debate on which is the best mesh to use in which patients in any specific situation became even more complex with the introduction of biosynthetic meshes to the market^24^. In this context, the collection of real-world data through registries may add evidence on the use of the different types of meshes in clinical practice, overcoming the generalizability or external validity of randomized controlled trials.

The present study provides data from an Italian registry on the use of biosynthetic meshes, Phasix and Phasix ST, which may represent possible alternatives to biologic prostheses in the case of complex abdominal repairs. Data on 75 patients with VHWG grade II or III and follow-up of at least 18 months showed the presence of minor complications. No deep or organ infections were reported, while 4% of patients reported a superficial infection requiring procedural interventions, and in one case (1.3%), the Phasix mesh was removed following an infection. Six patients (8%) had a recurrence. A seroma was reported in 17% of patients, but only five cases (6.7%) required percutaneous drainage. Most of these complications are comparable to data reported in a previous study derived from the same registry^15^. Although it utilized different selection criteria for the patients, that study reported 23.3% superficial infections, 16.3% seromas, 4.7% infected mesh removals, and no recurrences, deep infections or organ space infections.

In the present study, the recurrence rate of 8% may be explained by a longer follow-up and an increase in the number of cases. Phasix meshes are absorbable within 12–18 months^17^, and a follow up of at least 18 months can give us an idea of what happens once the prosthesis has been completely resorbed. Even though these data are very encouraging in terms of prevention of recurrences, a follow up of at least 3 years is needed to obtain reliable data, as reported by the Updated Italian Consensus Development Conference based Guidelines^25^. Data on a follow-up of 18 and 36 months have been presented by Roth and colleagues^26,27^; the latter study, which considered 82 patients with CDC Class I wounds (clean) treated with Phasix meshes, reported a hernia recurrence rate of 15.7%, a surgical site infection rate of 9.1% and a rate of seroma requiring intervention of 6.6%. A longer follow-up for the Italian Hernia Club, which is still registering cases, will be able to assess whether the complication rates are in line with these data.

Regarding quality of life, in the literature, there is a paucity of studies focusing on the QoL of patients after hernia repair and presenting utility coefficients for cost-effectiveness evaluations. Three studies presented data on patients with inguinal hernia who underwent repair with synthetic meshes^28–30^, while two other studies considered incisional hernia repairs with either synthetic^31^ or biosynthetic^32^ prostheses. To the best of our knowledge, no studies have provided health utility estimates for patients undergoing hernia repair with biological meshes. The first cited study^28^ compared open vs. laparoscopic groin hernia repair performed using mostly polypropylene tension-free meshes. The EQ-5D questionnaire was administered to patients at 1 week, 1 month and 3 months after the intervention. The authors found a significantly higher utility score in the laparoscopic group at week 1 (0.74 vs. 0.68), but a non-significant difference in the utility score at 3 months after the procedure (0.84 vs. 0.86). The second study^29^ assessed the health outcomes of laparoscopic inguinal hernia repair with synthetic heavy-weight (HWM) and lightweight meshes (LWM). The authors found a non-significant difference in 1-year health utility between HWM and LWM (0.91471 vs. 0.91377, respectively). The third study^30^ assessed the post-operative QoL of 90 patients undergoing groin hernia repair with Parietex ProGrip self-fixating mesh (Covidien, Dublin, Ireland), a synthetic mesh composed of monofilament polyester and polylactic acid grips. The EQ-5D questionnaire was administered at four time points after intervention: day 1, day 7, 1 month, and 6 months. The mean health utility increased significantly from 0.31 (day 1) to 0.95 at the end of the follow-up period.

Concerning QoL after incisional hernia repair, one study^31^ compared laparoscopic repair with anterior open repair. Eighty-four patients with incisional hernia were randomly allocated to an open group or to a laparoscopic group for hernia repair with a double-layer polypropylene–expanded polytetrafluoroethylene or standard polypropylene mesh, respectively. The EQ-5D questionnaire was administered on days 1, 2, 3, 5, 7, and 15 and at 1 month, 3 months and 1 year after the procedure. There were no significant differences in utility values between the two groups during the follow-up. Values ranged from 0.1682 (open group)—0.1136 (laparoscopic group) on day 1 to 0.9767 (open group)—0.9318 (laparoscopic group) at 1 year. Another observational study^32^ considered 104 patients with a clean-contaminated or contaminated wound (CDC criteria) undergoing ventral/incisional hernia repair with a different biosynthetic mesh (BIO-A, Gore). Quality of life and return of function were measured using the Short Form 12 Health Survey and the EQ-5D at baseline and post-operatively at day 30 and at 6, 12, and 24 months. This study reported utility values ranging from 0.73 at baseline to 0.84 at 24 months. Considering the same observation period, in our cases, the utility coefficients ranged from 0.768 to 0.974, showing a better QoL profile than that of the comparator biosynthetic mesh.

In the present study, data have been derived from a registry born from the spontaneous collaboration of Italian clinicians. Although several checks were made on the data before performing the statistics, and a systematic and comprehensive quality control procedure was performed to reduce measurement and input errors, some unavoidable input approximations were found. This was true especially for the clinical data, which were abstracted from the clinical report forms and copied into the registry, thus creating possible data discrepancies. Nevertheless, despite the imputation errors found, these did not weaken the analyses but indeed showed that the preliminary results were conservative.

Moreover, due to the different types of hernia, different techniques have been performed with diverse meshes positioning (e.g., inlay, sublay, onlay). So, the present study has limitations, and analyses considering different patients’ subgroups could be done in the future to provide more detailed results.

The present prospective multicentre observational study showed that Phasix biosynthetic meshes for the repair of abdominal hernia reported low hernia recurrence and post-operative wound infection rates, particularly in light of a patient population with VHWG grade II or III wounds. The use of a biosynthetic mesh can be an alternative for reducing costs and improve patients’ quality of life in the management of complex patients.

## Conclusions

The Italian Hernia Club registry represents, until now, the largest European registry containing real world data on hernia repair using Phasix meshes. The tool will give us the opportunity to monitor, in the future, real-world data regarding hiatal hernias, diastasis, parastomal hernias, stoma reinforcement and alternative mini invasive procedures (robotic and endoscopic approach), providing us with information on pathologies for which it is difficult to carry out high-level scientific studies.

Phasix meshes may be an alternative to synthetic and biologic prostheses in preventing recurrences; they have promising outcomes in terms of early and late complications and in improving patients’ quality of life. A longer follow up will be able to provide more consistent evidence.
